# MEK inhibitors enhance therapeutic response towards ATRA in NF1 associated malignant peripheral nerve sheath tumors (MPNST) *in-vitro*

**DOI:** 10.1371/journal.pone.0187700

**Published:** 2017-11-13

**Authors:** Susan Fischer-Huchzermeyer, Anna Dombrowski, Gordon Wilke, Verena Stahn, Anna Streubel, Victor Felix Mautner, Anja Harder

**Affiliations:** 1 Institute of Neuropathology, University Hospital Münster, Münster, Germany; 2 Clinics of Radiotherapy, HELIOS Clinic Berlin-Buch, Berlin, Germany; 3 Department of Neuropathology, Charité-Universitätsmedizin Berlin, Berlin, Germany; 4 Institute of Pathology, HELIOS Clinic Emil von Behring, Berlin, Germany; 5 Clinics and Polyclinics of Neurology, University Hospital Hamburg-Eppendorf, Hamburg, Germany; 6 Institute of Pathology, Medical University Brandenburg, Brandenburg, Germany; Laboratoire de Biologie du Développement de Villefranche-sur-Mer, FRANCE

## Abstract

**Objective:**

Neurofibromatosis type 1 (NF1) is a hereditary tumor syndrome characterized by an increased risk of malignant peripheral nerve sheath tumors (MPNST). Chemotherapy of MPNST is still insufficient. In this study, we investigated whether human tumor Schwann cells derived from NF1 associated MPNST respond to all-*trans* retinoic acid (ATRA). We analyzed effects of ATRA and MEK inhibitor (MEKi) combination therapy.

**Methods:**

MPNST cell lines S462, T265, NSF1 were treated with ATRA and MEKi U0126 and PD0325901. We assessed cell viability, proliferation, migration, apoptosis and differentiation as well as mRNA expression of *RAR* and *RXR* subtypes and ATRA target genes such as *CRABP2*, *CYP26A1*, *RARB* and *PDK1*. We also analyzed *CRABP2* methylation in cell lines and performed immunohistochemistry of human MPNST specimens.

**Results:**

ATRA therapy reduced viability and proliferation in S462 and T265 cells, accompanied by differentiation, apoptosis and reduced migration. NSF1 cells which lacked *RXRG* expression did not respond to ATRA. We furthermore demonstrated that ATRA signaling was functional for common targets, and that mRNA expression of *CRABP2* and its targets was raised by ATRA therapy, whereas alternative pathways via *FABP5* were not induced. Finally, combination of ATRA and MEKi demonstrated additively reduced viability of T265 and S462 cells.

**Conclusions:**

We observed therapeutic effects in two of three MPNST cell lines pronounced by combination therapy. These data point to a potentially successful treatment of MPNST by combined application of ATRA and MEK inhibitors such as U0126 or PD0325901.

## Introduction

Malignant peripheral nerve sheath tumors are soft tissue sarcomas that typically occur in the setting of Neurofibromatosis type 1 (NF1) [1, 2]. NF1 is a common hereditary tumor syndrome with a variable clinical expression [3]. The development of MPNST is the major cause of decreased life expectancy in NF1 patients, occuring in 8–13% of the patients over the life span [4]. NF1 associated MPNST develop from benign precursors, so called neurofibromas, the hallmark of the disease [5]. Whereas the first hit of the *NF1* gene explains some of the clinical signs, an additional loss-of-function mutation of *NF1* is required for the development of tumors, resulting in over activation of RAS signaling. NF1 associated neurofibromas exhibit individual second *NF1* hits, affecting precursor Schwann cells only [6–11]. Malignant transformation to MPNST is suggested to require accumulation of additional genetic aberrations. Most common molecular aberrations besides *NF1* inactivation are mutations in *RB1*, *SOX9* and *MET*, homozygous deletion of *CDKN2A*, *INK4A* and *ARF* tumor suppressors and loss of *TP53* [12–16]. Although different chemotherapeutic regimens have been applied and pre-clinical studies produced promising results, the outcome of patients with MPNST has not been significantly improved over the past decades. Thus, surgical resection is a standard treatment followed by chemotherapy. Although radiotherapy prolongs time to relapse, it does not improve survival [17, 18].

Using proteome analysis, we recently observed differential expression of the cellular retinoic acid binding protein 2 (CRABP2), transgelin (SM22)/TAGLN and eukaryotic translation initiation factor 4H (EIF4H) in subtypes of benign NF1-derived peripheral nerve sheath tumors [19]. TAGLN was shown to be involved in NF1 associated tumor progression via hypo-methylation and subsequent up-regulation and stimulation of MAPK signaling in MPNST [20]. Differential expression of CRABP2 was investigated due to its role in cellular transport of retinoic acid (RA) by our group in several NF1 derived tumors. In addition, ATRA independent functions were described [21]. We demonstrated presence of CRABP2 in neurofibroma derived Schwann cells, and therefore concluded that expression of CRABP2 might enable RA based therapeutic intervention in peripheral nerve sheath tumors [22–25]. Retinoic acid (RA) is a metabolic product of vitamin A, extracted from diet and stored as retinoid. Via binding to the retinol binding protein 4, RA is circulating through the blood and taken up by cells to be metabolized into all-*trans* retinoic acid (ATRA) [26]. In the cytoplasm ATRA binds to CRABP1 and CRABP2, whereat CRABP2 furthermore assists RA entering the nucleus. Here, RA associates with retinoic acid receptors (RAR) and retinoid X receptors (RXR) that bind specific genomic regions called retinoic acid response elements (RARE) to allow transcription. The activation of ATRA mediated signaling pathways is also possible via binding of the fatty acid binding protein 5 (FABP5) to the peroxisome-proliferator-activated receptor (PPAR) / RXR nuclear complex. The intracellular ratio of CRABP2 and FABP5 protein expression is crucial for the activation of either of the two pathways [27]. ATRA is a regulator in embryonic development, especially in patterning and neuronal differentiation and furthermore essential for adult tissue homeostasis, neuronal plasticity and signal transduction of retina [28]. Due to its anti-carcinogenic activities ATRA is used in therapy and prevention of cancer such as acute promyelocytic leukemia (APL) and acute myeloic leukemia (AML) [29, 30]. *In-vitro* and *in-vivo* effects of ATRA therapy were also seen in cervical cancer, lung cancer, colon adenocarcinoma, breast cancer, kidney cancer, neuroblastoma, germ cell tumors, and glioblastoma [31–36]. However, clinical use of ATRA can be hampered by retinoic acid resistance. Hence, ATRA is used in combination therapy utilizing potential synergistic effects. Administration of 13-*cis* RA significantly improved overall survival after consolidation therapy of neuroblastoma in children, and patients with relapse of APL showed a lower relapse rate when ATRA was combined with anthracycline [37, 38]. Benefits of combination therapy were also shown for metastatic renal cell carcinoma (13-*cis*-RA + IFNA2) and acute myeloic leukemia (ATRA + valproic acid) [39, 40]. In neuroblastoma cells, being resistant to ATRA due to loss of the zinc finger protein ZNF423, the inhibition of the MAPK cascade restored ATRA responsiveness. ZNF423 loss was addressed to *NF1* loss that in turn was supposed to cause the over activation of the Ras-MEK signaling pathway in these cells [41, 42].

Here, we investigated if treatment of tumor cells derived from NF1 associated MPNST respond to ATRA therapy. We furthermore analyzed, if combination of ATRA and MEK inhibitors (MEKi) enhance therapeutic effects. Thus, we elucidated involved signaling pathways and molecular mechanisms of ATRA in MPNST cells. Finally, our *in-vitro* data point to a very promising therapeutic approach for human MPNST by combining ATRA and MEKi application.

## Materials and methods

### Primary cell cultures and cell lines

Normal primary human and rat Schwann cells, the MCF-7 cell line and three human NF1 associated MPNST cell lines (S462, T265, NSF1) were analyzed [43–47]. MPNST cell lines S462 and T265 were provided by VF Mautner (University of Hamburg). The NSF1 cell line was provided by D Kaufmann (University Hospital Ulm, Germany). The MCF-7 cell line was provided by V Senner (University Hospital Muenster, Germany). Experiments were performed in cooperation with VF Mautner and were approved for these purposes by ethics committee of „Ärztekammer Hamburg” (19.04.2011, No. WF-018/11). Normal primary rat Schwann cells (nrSC) were provided by B Gess (University Hospital Muenster, Germany). Primary normal human Schwann Cells (nhSC) were purchased from ScienCell (HSC, California, USA). Primary Schwann cells were cultivated in standard Dulbeccos modified eagles medium (DMEM) containing 4.5 g/L glucose, 2 mM L-glutamine, 10% fetal bovine serum (FBS), 1 mM sodium pyruvate and 100 U/mL penicillin/streptomycin supplemented with 0.5 mM 3-iso-butyl-1-methylxanthine (IBMX), 10 nM β1-heregulin, 2.5 μg/mL insulin and 2.5 mg/L fungizone. Culture vessels were coated with 1 mg/mL poly-L-lysine (10 min at room temperature) and 4 μg/mL laminin/PBS (2 hours at 37°C). Enrichment of Schwann cells was achieved by mild trypsination and adhesion of fibroblasts to uncoated culture vessels. All primary cultures showed a proportion of Schwann cells (versus fibroblasts) of above 95% (S100 immunostaining). MPNST cell lines and the MCF-7 cell line were cultured using standard DMEM in uncoated vessels.

### Treatment of cells with ATRA and MEK inhibitors (MEKi)

ATRA (Sigma-Aldrich), MEK inhibitors U0126 (NEB) and PD0325901 (Selleckchem) were dissolved in DMSO, aliquots were stored at -80°C. Treatment was performed for 7 days unless otherwise stated (three to five replicate wells per sample) (Table A in S8 Fig). Control cells were treated with medium containing 0.1% DMSO. Medium supplemented with pharmaceuticals was changed every second day. Cells under treatment were protected from light by aluminum foil. Experiments were performed in triplicates at least.

### RNAi by transient transfection of siRNA

Transient transfection of siRNA (12 nM) was performed using HiPerFect reagent (Qiagen) according to the manufacturer’s protocol. Simultaneously to transfection with specific siRNA, a non-specific siRNA (si-scrambled) was used as control. Cells were seeded the day before transfection.

### Proliferation and migration assays

Cell proliferation and migration of cells was analyzed by xCELLigence DP system (Roche) [48, 49]. Migration and proliferation of cells were detected by measurement of impedance of gold electrodes, that refers to “Cell Index”. Migration assays were performed in CIM-plates^®^ according to the manufacturer’s instructions and monitored for 24 h. For analysis of migration after ATRA treatment, cells were pre-treated with ATRA (5 μM) / DMSO for two days before seeding for migration analysis under treatment conditions. Cell proliferation was monitored continuously in E-plates^®^ for 120 h according to the manufacturer’s instructions. All experiments were performed in triplicates with 4 replicates.

### Viability assay

Viability of cells was determined using the 3-(4,5-dimethylthiazol-2-yl)-2,5-diphenyl-tetrazolium bromide (MTT)-assay in 48- or 96-well plates. Cells were incubated with 0.5 g/L MTT labelling solution / medium for 2 or 3 hours, depending on cell line, Thereafter, medium was aspirated and cells were incubated with isopropanol for 5 min on an orbital shaker at 180 rpm. Measurement of absorbance was performed at 560 nm and at the reference wavelength of 750 nm in a GloMax^®^-Multi+ Detection System (Promega).

### Apoptosis (TUNEL) assay and flow cytometry

Cells treated with 5 μM ATRA (7 d) were subjected to APO-BrdU TUNEL Assay kit (Invitrogen) according to the manufacturer’s instructions for immune fluorescence and flow cytometry applications. For analysis of differentiation, cells were labeled with anti-S100 or anti-PMP22 antibodies and Alexa Fluor 488 secondary antibodies. Cells treated similarly but without primary antibody served as internal control. Cells were fixed in 1% ice-cold paraformaldehyde (PFA) and permeabilized in 70% ethanol before labeling with primary antibodies. Labeled cells were analyzed using a BD FACS Canto II. Each flow cytometry analysis was performed on 10.000 cells and experiments were performed 2–3 times.

### Isolation and quantification of RNA and DNA

Total RNA from cultured cells was isolated using Gene Elute Mammalian total RNA Miniprep Kit (Sigma) according to the provided protocol including DNase digestion with the RNase-free DNase Set (Qiagen). Isolation of genomic DNA from cultured cells was performed using Quick-gDNA MiniPrep Kit (Zymo Research) according to the provided protocol. Concentration of RNA and DNA was determined by using a ND-1000 spectrophotometer (NanoDrop Technologies) using ND-1000 V3-8.4 software.

### Polymerase Chain Reaction (PCR)

CDNA was prepared from 100–500 ng of total RNA with oligo (dT) primers and High Capacity cDNA-RT Kit including RNase inhibitor (Applied Biosystems) according to the manufacturer’s instructions. PCR was performed in a PTC-200 Peltier Thermal Cycler (MJ Research) using the HotStarTaq DNA Polymerase Kit (Qiagen). Each preparation contained 10 x PCR Buffer, 2 mM MgCl_2_, 200 μM of each dNTP (dNTP Mix, PCR Grade, Qiagen), 1 U Taq Polymerase, 0.4 μM of each primer (Table B in S8 Fig) and 1 μL cDNA template corresponding to 1–25 ng total RNA applied in cDNA-synthesis. Amplified cDNA fragments were separated in agarose (1.5%, 10 μg/mL ethidium bromide) and visualized in a Gel Doc EZ Imager (BioRad). Quantitative real-time-PCR (qRT-PCR) was performed in a StepOnePlus real time PCR system (Applied Biosystems). Endogenous GAPDH (glyceraldehyde 3-phosphate dehydrogenase) served as internal control. Expression levels of specific mRNA sequences were calculated relative to GAPDH levels using the ΔΔCT method described previously [50]. QRT-PCR using TaqMan Universal Master Mix (Applied Biosystems) was performed in a total volume of 20 μL, containing 1 μL Gene Expression Assay (Applied Biosystems) or 0.4 μM of each primer (Table C in S8 Fig) and 1 μL cDNA template. QRT-PCR using SYBR Green PCR Master Mix (Applied Biosystems) was performed in a total volume of 15 μL, containing 0.2 μM of each primer (Table B in S8 Fig) and 1 μL cDNA template.

### Methylation analysis of the CRABP2 promoter

Sodium bisulfite treatment of DNA was performed using the EpiTect^®^ Fast DNA Bisulfite Kit (Qiagen) according to manufacturer’s instructions. Primers (Tables C and D in S8 Fig) were designed using the PyroMark Assay Design 2.0 software (Qiagen). PCR was performed using the Maxima Probe qPCR Master Mix including Maxima Hot Start Taq DNA Polymerase (Thermo Fisher Scientific) in a total volume of 20 μL, containing 0.5 μM of each primer. Pyro-sequencing was performed in a PyroMark Q24 system (Qiagen) according to the manufacturer’s instructions, using the PyroMark Gold Q24 Reagents Kit (Qiagen). Fully methylated DNA and un-methylated DNA were used as controls. In order to visualize successful bisulfite conversion a T was added into the dispensation order. Quantification of CpG sites was performed with PyroMark Q24 software (Qiagen).

### Western blot analysis

To obtain whole cell protein extracts, cells were washed twice with cold PBS and lysed with RIPA buffer containing protease inhibitors. Determination of protein concentrations was performed by using the Lowry method and the DCTM Protein Assay Kit (BioRad) according to the manufacturer’s instructions. The absorbance at 750 nm was detected in a GloMax^®^-Multi+ Detection System [51]. Protein samples were separated by SDS-PAGE according to the Laemmli method at 120 V until the desired separation had been reached (1–1.5 h). To determine molecular weight of detected protein bands a prestained protein marker (Precision Plus Protein^™^ Dual Color Standards, BioRad) was loaded [52]. A tank-blotting chamber (Mini Trans Blot Cell, BioRad) was used to transfer proteins to a PVDF membrane (Roche) at 250 mA for 45 min. After transfer, the PVDF membrane was incubated with blocking solution followed by incubation with primary antibodies (Table E in S8 Fig) overnight at 4°C. Afterwards, the membrane was incubated in blocking solution, containing a hrp-conjugated secondary antibody (Table E in S8 Fig), at room temperature for 1–2 h. Finally, the membrane was incubated with Luminata^™^ Forte Western HRP Substrate (Millipore) to quantify signals by the Chemi Doc MP Imaging system (BioRad) using Image Lab^™^ software (BioRad).

### Immunohistochemistry and immunocytochemistry

Human MPNST specimens from NF1 patients and non-NF1 patients were kindly provided by Prof. W. Paulus (Institute of Neuropathology, University Hospital Muenster, Germany). These experiments were approved by ethics committee at „Ärztekammer Westfalen-Lippe Münster”(2007-261-f-S). Written informed consent was provided for participation. MPNST cell pellets were frozen at -80°C, fixed in 4% PFA and stored in 70% ethanol until paraffin embedding. Hematoxylin and eosin staining (HE) of FFPE samples was performed in an automated staining instrument Tissue Tek Glas and Prisma system (Sakura). For immunohistochemistry of FFPE samples, slices were deparaffinised and pre-treated with target retrieval solution (pH 6.1). Immunohistochemistry was performed using the DAKO REAL^™^ Detection Kit (K5001) according to the provided protocol and the automated staining instrument AutostainerLink 48 (Dako). Deparaffinised sections were incubated with primary anti-CRABP2 antibody (Abcam ab181255, monoclonal, from rabbit, 1:100). Staining was visualized by DAB staining followed by nuclear counterstaining with hematoxylin.

### Statistical analysis

We used unpaired t-test, one-sided t-test, Wilcoxon test, Mann-Witney-U test, Kruskal-Wallis test and Students t-test for analyses. A p-value of < 0.05 was considered to be significant.

## Results

### MPNST cell lines S462 and T265 are sensitive to ATRA treatment

**Fig 1:**
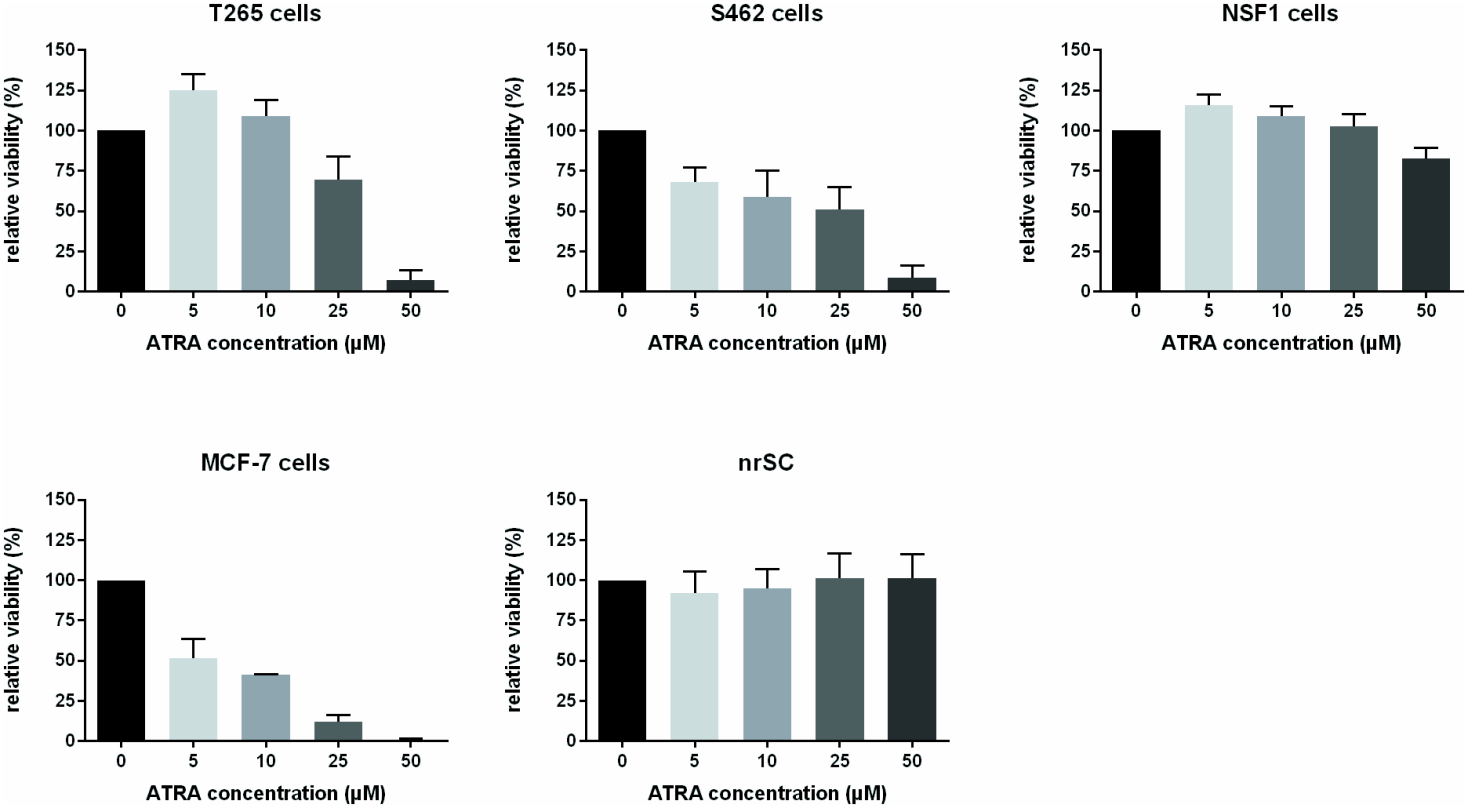
ATRA therapy affects viability of MPNST cells in a dose-dependent manner. Viability of cells under ATRA treatment was monitored by MTT assay on day 7 of treatment. T265 cells showed reduced viability at high ATRA concentrations (25 μM and 50 μM) and enhanced viability at low ATRA concentrations (5 μM and 10 μM). S462 cells demonstrated reduced viability at all ATRA concentrations. MCF-7 cells used as control were sensitive to ATRA treatment at all concentrations. In contrast, normal rat Schwan cells displayed no change of viability under ATRA treatment. Bars represent mean values of 3–4 repeated measurements within each cell line.

We investigated if MPNST cells (NSF1, S462, T265) were sensitive to ATRA treatment. We first determined expression of *RAR* and *RXR* subtypes in MPNST cells by RT-PCR, normal human Schwann cells and fibroblasts, since intact expression of nuclear receptors is an essential requirement for mediation of ATRA effects: All receptor subtypes were expressed in normal Schwann and MPNST cells, except of the *RXRG* subtype which was absent in NSF1 cells (S1 Fig). We then investigated effects of ATRA therapy on viability at day 7 of treatment: A dose-dependent decrease of viability was seen in T265 and S462 cells, whereas NSF1 cells did not respond (Fig 1). Normal primary rat Schwann cells as well as ATRA sensitive MCF-7 cells were used as controls. MCF-7 cells were sensitive to treatment with ATRA, whereas normal rat Schwann cells were not (Fig 1). By pyrosequencing, we analyzed, if methylation status of the *CRABP2* promoter region influenced response to ATRA treatment. In MPNST as well as in control cells methylation analysis of three different promoter regions of *CRABP2* (S2A Fig) did neither reveal a correlation of ATRA sensitivity with a specific methylation pattern (S2B Fig) nor an influence of ATRA therapy on promoter methylation (S2C Fig). Nevertheless, expression of CRABP2 in MPNST cell lines increased with decreasing promoter methylation.

In conclusion, a markedly reduced viability and proliferation at concentrations > 10 μM was seen in 2/3 MPNST cell lines under ATRA treatment, indicating ATRA sensitivity in a subset of malignant tumor derived Schwann cells.

### ATRA treatment induces morphological changes and differentiation

**Fig 2:**
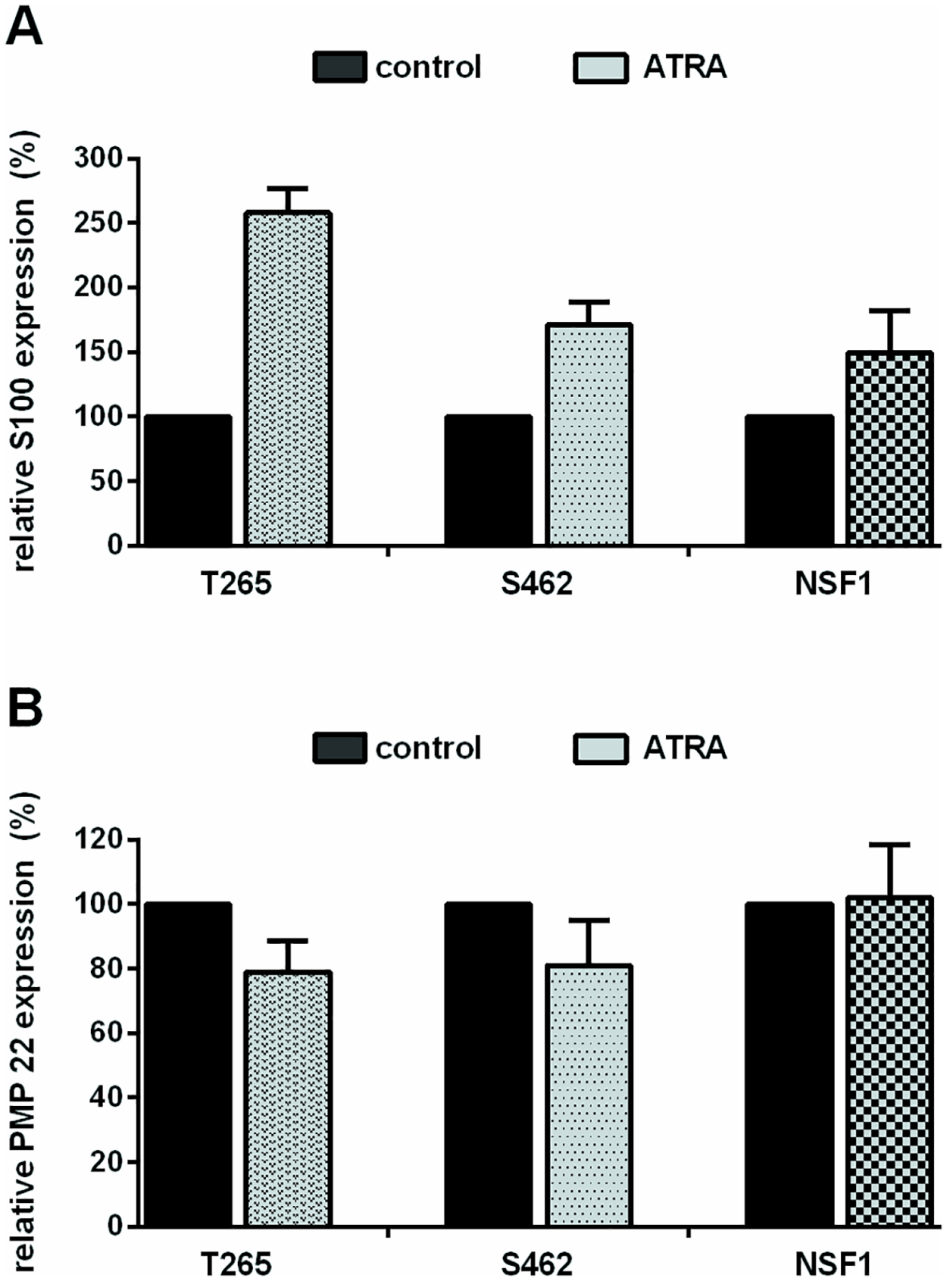
ATRA treatment induces expression of S100 and inhibits PMP22 expression. Flow cytometry analysis (FACS) of S100 and PMP22 protein expression in ATRA treated MPNST cells on day 7 of treatment (5μM). Bars indicate increase or decrease of fluorescence intensity (%) compared to untreated controls (100%). Values represent mean values (± SD) of 2–4 repeated measurements within each cell line: T265 cells showed an increase of 158% ± 19%, S462 cells of 71% ± 18% and NSF1 cells of 49% ± 33% (A). T265 cells showed a reduction of PMP22 expression of 22% ± 10%, and S462 cells of 20% ± 14% (B).

FACS analysis was carried out to analyze potential ATRA mediated differentiation of cells. The proportion of PMP22- and S100-positive Schwann cells after treatment was assessed (Fig 2): Expression of S100 protein increased in all MPNST cells. A reduction of PMP22 expression was seen in 2/3 MPNST cell lines (T265, S462). Next, morphological changes resulting from ATRA treatment were assessed by FACS: all MPNST cell lines revealed significantly higher mean values concerning relative cell size (forward scatter) as well as granularity (sideward scatter) in treated versus untreated cells (S3 Fig).

To summarize, anti-proliferative properties of ATRA in MPNST cell lines are accompanied by morphological changes and Schwann cell differentiation.

### ATRA treatment reduces migration and induces apoptosis

**Fig 3:**
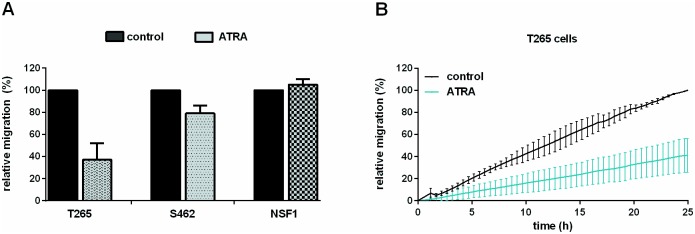
ATRA treatment reduces migration in 2/3 MPNST cell lines. Migration of MPNST cells under ATRA treatment, monitored using the xCELLigence system. Area under curve (AUC) was calculated after 16 h. Bars indicate AUC values (mean of 3–5 repeated measurements within each cell line) compared to control cells. T265 cells (25 μM ATRA) showed strongly reduced migration down to 37% ± 15%. A minor effect of ATRA treatment was observed in S462 cells (10 μM ATRA) with reduced migration of 79% ± 7%. No effect on migration was observed in NSF1 cells (50 μM ATRA) (105% ± 5%) (A). Migration curves of ATRA treated T265 cells (ATRA, grey line) and untreated T265 cells (control, black line) are demonstrated exemplarily (B).

**Fig 4:**
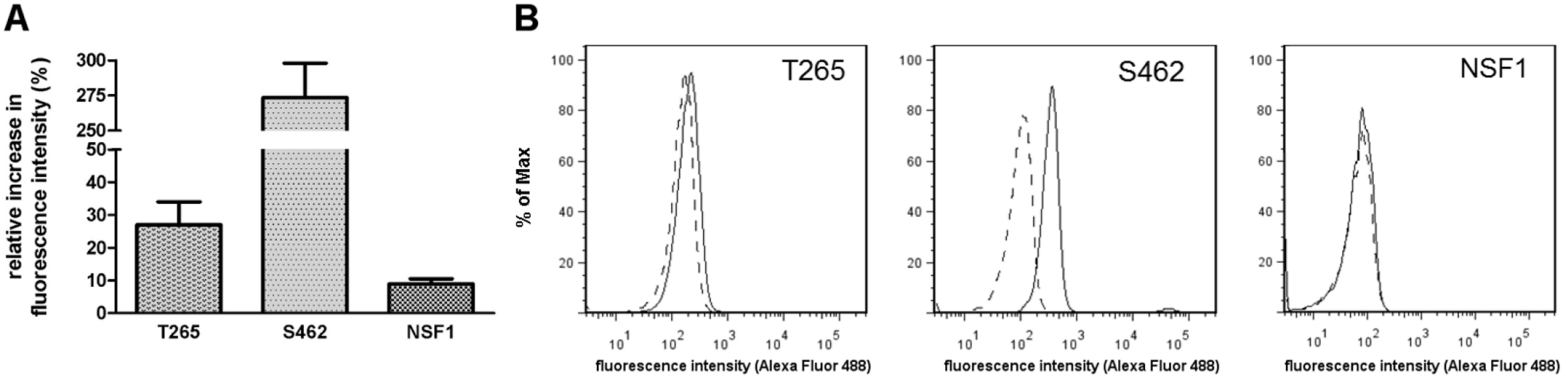
ATRA treatment induces apoptosis in T265 and S462 cells. Flow cytometry analysis (FACS) of TUNEL staining in ATRA treated MPNST cells at day 7 (5 μM). Bars represent intensity values (mean of 2–3 repeated measurements within each cell line) compared to control cells (black bars): Increase of TUNEL staining intensity to 127% ± 10% in T265 cells, to 374% ± 35% in S462 cells and 109% ± 3% in NSF1 cells (A). Right shift (regular line) in fluorescence intensity compared to untreated controls (dashed line) in the FACS histogram is shown for T265, S462 and NSF1 cells (B).

To evaluate migration as a parameter for tumor invasion, we monitored MPNST cell lines under ATRA therapy in real-time (xCELLigence system). Whereas NSF1 cells did not show reduced migration due to ATRA therapy, a strong inhibition of migration was observed in cell line T265 and a minor effect was seen in cell line S462 (Fig 3). To evaluate induction of apoptosis, we performed TUNEL staining and a FACS based TUNEL assay: Increase of apoptosis was seen in T265 MPNST cells as the proportion of TUNEL positive cell nuclei increased in ATRA treated cultures compared to untreated controls (S4 Fig). A FACS based TUNEL assay demonstrated pro-apoptotic effects in 2/3 MPNST cell lines treated with ATRA: The strongest effect was seen in S462 cells showing an increase to 374% ± 35% in fluorescence intensity compared to untreated cells (Fig 4).

In summary, 2/3 MPNST cell lines showed reduction of migration due to ATRA treatment. Anti-proliferative properties of ATRA on tumor Schwann cells are mediated by induction of apoptosis.

### ATRA therapy induces expression of target genes

**Fig 5:**
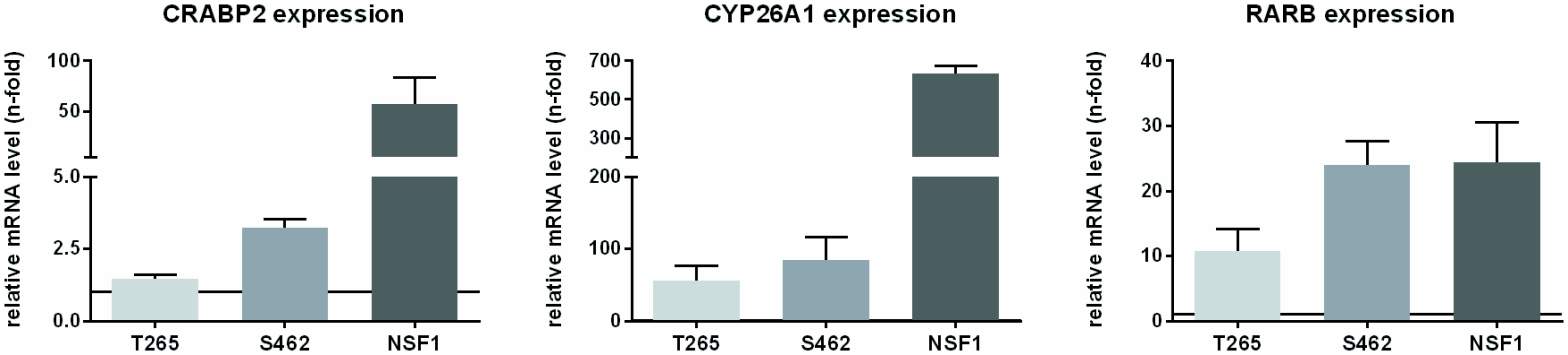
ATRA treatment induces expression of classical targets in MPNST. Relative mRNA expression of bona fide ATRA targets was analyzed in MPNST cells after ATRA treatment by qRT-PCR. Bars represent levels of *CRABP2*, *CYP26A1* and *RARB* relative to untreated control cells (black line, 1-fold). All three MPNST cell lines showed induction of the investigated targets. T265 cells showed lowest induction (*CRABP2*, *CYP26A1*, *RARB*) by ATRA treatment (1.5-fold ± 0.1, 55.8-fold ± 21.1, 10.8-fold ± 3.3) whereas NSF1 cells showed highest induction of mRNAs after ATRA treatment (57.8-fold ± 26.2, 633.0-fold ± 43.6, 24.5-fold ± 6.1) among the three MPNST cell lines. Measurements were repeated in each cell line 3 times.

To identify if ATRA signaling was functional in MPNST cell lines treated with ATRA, we analyzed expression of bona fide CRABP2 targets such as *CRABP2* itself, *CYP26A1* and *RARB* by qRT-PCR. Promoters of these target genes contain a RARE element and are therefore inducible by ATRA [53]. We found all targets to be induced in cells treated with ATRA confirming a transcriptional activation (Fig 5). Since ATRA is known to mediate effects not only via CRABP2 and RXR/RAR but also via FABP5 and PPAR/RXR (both reflecting opposing signaling pathways), we analyzed expression of *FABP5* and its signaling target *PDK1*. In 3 MPNST cell lines investigated by qRT-PCR, *FABP5* and *PDK1* mRNA levels were not elevated (S5 Fig).

As CRABP2 is the major mediator of cellular ATRA response, its expression in a series of tumor Schwann cells had been analyzed in previous studies [21]. Here we demonstrated that Schwann cells in human MPNST (FFPE) as well as MPNST cell lines expressed CRABP2. To conclude, ATRA signaling was shown to be functional for targets of CRABP2 mediated ATRA signaling pathways. During ATRA therapy, mRNA expression of *CRABP2* raised, whereas alternative pathways (via FABP5 and subsequent *PDK1* up-regulation) were not induced.

### MEK inhibitor treatment reduces viability

**Fig 6:**
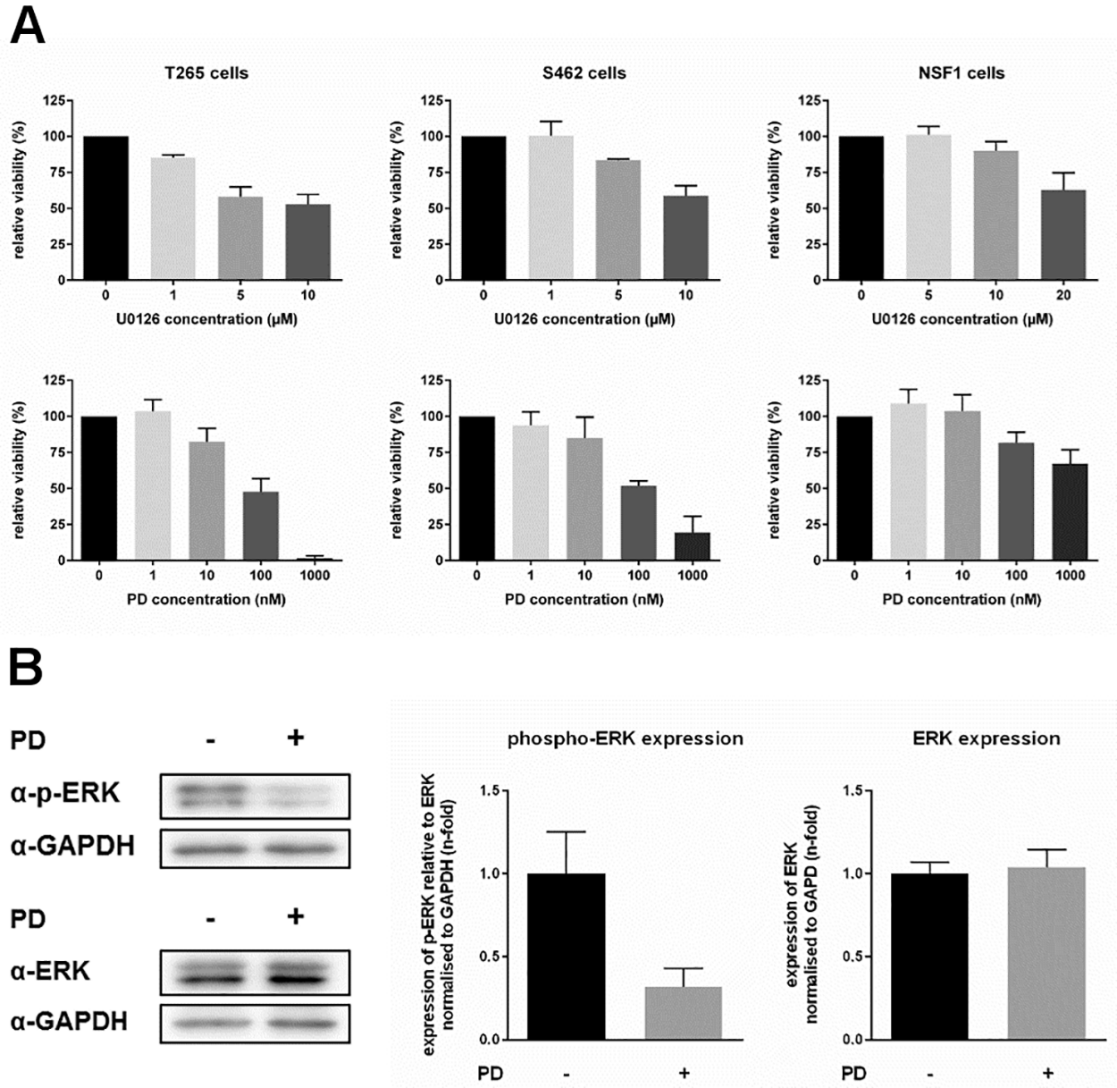
MEK inhibition reduces viability of MPNST cells. Viability of MPNST cells under MEKi treatment was monitored by MTT. MPNST cells were treated with different doses of MEKi U0126 (upper panel) or PD0325901 (lower panel). T265 cells and S462 cells highly responded to MEKi treatment with reduced viability, whereas NSF1 cells did not show enhanced sensitivity to MEKi treatment, compared to untreated control cells (black bars). Maximum reduction of viability was achieved at 10 μM of U0126 and 1 μM of PD0325901 (reduction down to 53% ± 7% and 2% ± 1% in T265 cells, and 59% ± 7% and 19% ± 11% in S462 cells) (mean value of 3–5 measurements in each cell line (A). Relative ERK-P expression in T265 cells after MEKi treatment was analyzed. Western blots of ERK-P, ERK and GAPDH antibodies are demonstrated exemplarily for T265 cells after treatment with 10 nM PD0325901 (+) compared to untreated cells (-). T265 cells treated with MEKi demonstrated strongly reduced phosphorylated ERK level (grey bar, 0.32-fold ± 0.11-fold) compared to untreated control cells (black bar, 1.00-fold ± 0.25-fold). PD0325901 treatment did not affect total ERK level in treated (grey bar, 1.04-fold ± 0.07-fold) versus untreated T265 cells (black bar, 1.00-fold ± 0.11-fold) (mean value of 3 repeated measurements).

We first analyzed exclusive MEKi administration. MEKi treatment is known to decrease NF1 associated overactive MAPK signaling and to restore retinoic acid sensitivity in NF1 lacking neuroblastoma cells [41, 42]. Therefore, we determined the effects of MEKi U0126 and PD0325901 treatment on viability of cells (MTT-assay): A dose dependent response was achieved by treatment with MEKi U0126 in MPNST cell lines T265, S462, and NSF1 (Fig 6A), although effectiveness was much lower in NSF1 cells. Treatment of tumor cells with MEKi PD0325901 revealed a reduction of viability in T265 and S462 cells at lower doses, whereas NSF1 cells did not show marked sensitivity (Fig 6A). At 1000 nM viability of T265 cells was reduced to 2% ± 1% and viability of S462 cells was reduced to 19% ± 11%. NSF1 cells showed a slight reduction to 67% ± 10%.

To substantiate downstream effects of MEKi, we analyzed expression of phosphorylated ERK (ERK-P). Elevated levels of Ras-GTP and activation of ERK is typical for NF1 associated MPNST. In principal, ERK phosphorylation is mediated by MEK1/2, which can be inhibited by MEKi such as PD0325901. Since reduced ERK-P expression has already been shown for human S462 MPNST cells after treatment with PD0325901 [54] we here tested ERK-P in T265 cells as a proof of principle for our experiments: ERK1/2 phosphorylation was significantly reduced in treated versus untreated T265 MPNST cells (Fig 6B). Additionally, we analyzed a transcriptional co-activator for retinoic acid receptors, ZNF423, which has been described to be lost in human NF1 lacking neuroblastoma cells. Absence of ZNF423 was demonstrated to explain decreased retinoic acid response which was restored by MEKi treatment [41, 42]. In contrast to these findings, we did not observe any induction of *ZNF423* after PD0325901 treatment in all NF1 deficient MPNST cell lines. Unexpectedly, *ZNF423* levels decreased under PD0325901 administration in T265 and NSF1 cells (S6 Fig).

To summarize, MEKi treatment reduced viability in 2/3 MPNST cell lines and was shown to be functional since ERK1/2 phosphorylation was reduced. Contrarily to previous reports, *ZNF423* levels decreased or were not affected at all by MEKi treatment.

### ATRA administration boosts impaired viability resulting from MEKi therapy

**Fig 7:**
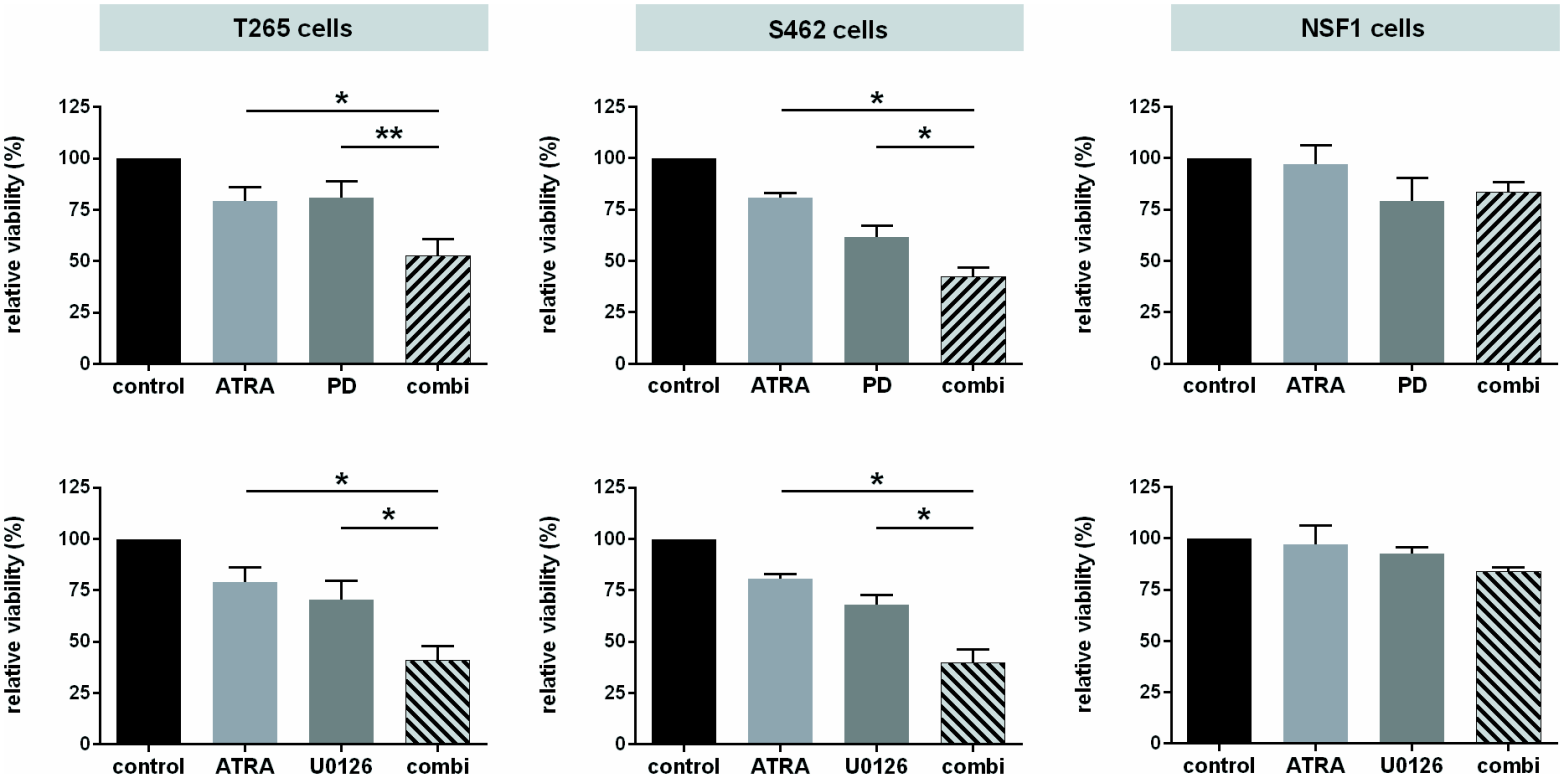
Combined ATRA and MEKi treatment adds reduced viability. Relative viability of MPNST cells after combination therapy (day 5) was assessed by MTT assay. MPNST cells were treated with ATRA (light grey) and MEKi (dark grey) alone or in combination (striped). PD0325901 combination treatment is shown in the upper panel, U0126 combination treatment in the lower panel. A significant reduction of viability in the combination therapy group was found for T265 cells and S462 cells with both inhibitors, compared to mono-treated cells. Bars represent mean values of repeated (3–5 times) measurements in each cell line). NSF1 cells displayed no additive effects due to combination-therapy, neither with PD0325901 nor with U0126, as well as a very weak response to monotherapy.

Wecombined ATRA and MEKi treatment. Compared to monotherapy, combined therapy revealed a significant reduction of viability of T265 and S462 cells in an additive manner using both U0126 and PD0325901 (Fig 7). For example, administration of 10 nM PD0325901 and 10 μM ATRA reduced viability of T265 cells to 53% ± 8%, compared to 79% ± 7% under ATRA monotherapy or 81% ± 8% under MEKi monotherapy. NSF1 cells were not sensitive to mono- or combination therapy. Finally, we examined *CRABP2*, *CYP26A1* and *RARB* gene expression after mono- and combination therapy by qRT-PCR. Bona fide targets of ATRA-mediated signaling were induced in all MPNST cell lines, both under mono- and under combination therapy. Here, additive effects due to combination treatment were not seen (S7 Fig).

In conclusion, combined treatment of ATRA and MEKi induced expression of bona fide targets of ATRA mediated signaling such as *CRABP2*, *CYP21A1*, and *RARB*. The combination of ATRA and MEKi U0126 or PD0325901 provokes additive effects on reduced viability of T265 and S462 cells, suggesting a new and potentially successful therapeutic option for MPNST.

## Discussion

MPNST display aggressive and invasive growth and an early onset of metastasis [42]. Patients have a poor prognosis with 5 year survival rates between 26–60% [43–46]. About 40% of MPNST are associated with NF1, warranting regular checkup in NF1 patients with a high tumor load [47]. In our study we could demonstrate that *in-vitro* combination of ATRA and MEKi reduces proliferation of tumor Schwann cells derived from NF1 associated MPNST. Reduced proliferation by ATRA administration alone, seen in MPNST cell lines T265 and S462, was markedly intensified by combination with MEKi U0126 or PD0325901. MEKi treatment alone reduced viability in T265 and S462 MPNST cells accompanied by reduced ERK1/2 phosphorylation. Further studies should address if these combined effects are also achieved at doses of ATRA that are used for clinical therapy such as 1–2 μM. Compared to benign neurofibromas MAPK signaling is further elevated in MPNST cells which contributes to their aggressive malignancy. MAPK inhibition seems to further repress tumor cell survival combined with ATRA treatment. Our results indicate a therapeutic potential of ATRA. Combination with MEKi constitutes a novel approach for NF1 associated malignant peripheral nerve sheath tumors.

Interestingly, ATRA was shown to display anti-proliferative properties by induction of apoptosis, differentiation and morphologic changes and by reduction of cell migration (Table 1). ATRA sensitivity remarkably differed between the cell lines with most pronounced effects in S462 and T265 cells, which warrant further investigations. We demonstrated that ATRA induces moderate anti-proliferative effects in MPNST cells T265 and S462 via apoptosis. These findings are in concordance with other studies describing induction of apoptosis via increased expression of pro-apoptotic genes such as *CASP7* and *CASP9* in MCF-7 cells. Interestingly, *CASP9* contains a functional RARE which implicates this gene to be a direct target of CRABP2-mediated ATRA signaling [33]. Reduced viability of MPNST cells treated with ATRA was accompanied by reduced migration properties and signs of differentiation. The latter was also recently shown for neuroblastoma cells, underlining that induction of differentiation and pro-apoptotic signaling by retinoic acid are the reasons for its general usage in oncology and dermatology [55, 56]. We think that apoptosis that we measured at day 7 after treatment may not largely influence migration of cells in our assay which was performed within the first 24h after treatment. To completely rule out an influence an apoptosis on migration behavior an apoptosis inhibitor might be applied when measuring migration of MPNST cells in further experiments. In fact, in agreement with our results, an earlier study described induction of morphological changes in MPNST cells from a spindle-cell to an epitheloid-cell phenotype using ATRA. [57]

**Table 1:** Therapeutic pattern and gene expression profiles in MPNST cells. Relative expression, functional effects and promoter methylation is depicted in a semi-quantitative manner applying the following score: three symbols—highest, two symbols—medium, one symbol—low, dash—no, induction/reduction/expression among the three MPNST cell lines.

	T265	S462	NSF1
**CRABP2 promoter methylation**	-	+++	+
**RAR (A, B, G) and RXR (A, B, G) mRNA expression**	all expressed	all expressed	RXRG not expressed
**ATRA treatment**			
CYP26A1 / RARB / CRABP2 mRNA level	↑	↑↑	↑↑↑
PDK1 mRNA level	-	-	-
Viability	↓↓	↓↓↓	-
Proliferation	↓↓	↓↓↓	-
Migration	↓↓↓	↓	-
Apoptosis	↑↑	↑↑↑	-
**MEKi treatment**			
Viability	↓↓↓	↓↓	↓
**Combination treatment**			
Viability	↓↓↓	↓↓↓	-
**Sensitivity towards treatment**	**+++**	**+++**	**-**

In our hands, NSF1 MPNST cells demonstrated resistance to ATRA therapy since no major effects were seen regarding viability, proliferation, apoptosis and migration under ATRA treatment. However, loss of *RXRG* expression identified in NSF1 cells seems not to explain resistance to ATRA entirely, due to the fact that we demonstrated these cells to highly upregulate *CRABP2* and its downstream targets after ATRA administration (Table 1). In contrast, embryonic stem cells lacking RARG fail transcriptional activation of ATRA target genes such as *CRABP2* and *CYP26A1* after ATRA treatment [58]. Although little is known about the role of RXRG in Schwann cells, absent expression of *RXRG* in NSF1 cells may contribute to resistance to ATRA therapy through other mechanisms. Similarly, a defective *RARB* promoter was demonstrated to lead to partial insensitivity of thyroid cancer cells [59, 60]. A general pre-therapeutic testing of elements necessary for a functional retinoic acid transcription complex may uncover ATRA resistant MPNST subtypes prior to therapy. Another explanation for resistance of MPNST to ATRA treatment would include *CRABP2* promoter methylation as a determinant of RA effects [56, 61]. We expected *CRABP2* methylation being causal in those cells that exhibited worse response to ATRA. Although we covered three different promoter regions between -445 up to +370 relative to TSS, we failed to determine a correlation between ATRA sensitivity and *CRABP2* promoter methylation as it was shown for other cancer cells such as for gliomas, medulloblastomas, Wilms tumors and pancreatic adenocarcinomas [56, 61–63].

Since CRABP2 mediates functions of ATRA, we investigated *CRABP2* expression and response to RA signaling *in-vitro* in detail. Bona fide targets of CRABP2-mediated ATRA signaling such as *CRABP2*, *RARB*, and *CYP21A1* were demonstrated to be up-regulated due to ATRA treatment in all MPNST cell lines (Table 1) as it was shown in other studies for mouse embryonic carcinoma cells, for COS-7 monkey fibroblast cells as well as for head and neck squamous cell carcinoma cells [64–66]. Although ATRA treatment induced common targets in MPNST cell lines, functional effects of treatment were not seen in NSF1 cells, pointing to other resistance mechanisms as discussed above. Since ATRA is able to induce also opposing effects via FABP5 [27], we furthermore investigated mRNA of its target *PDK1* in MPNST cells. We demonstrated that expression levels of *PDK1* were not affected under ATRA treatment in all MPNST cell lines. The ratio of FABP5/CRABP2 was previously demonstrated to be associated with ATRA resistance in gliomas, with prognostic behavior in pancreatic ductal adenocarcinoma cell lines, with activation of PPAR instead of RAR in a breast cancer mouse model and with tumor grade and prognosis in breast cancer [63, 67–69]. In our study, we verified that FABP5 level did not interfere with ATRA sensitivity in MPNST, like in other human cancers. Thus, CRABP2 is not only necessary to enable ATRA signaling but is the prevalent mediator compared to opposing signaling pathways in MPNST. To underline these findings, we were able to detect expression of CRABP2 in a series of human MPNST FFPE specimens and in all three MPNST cell lines. Thus, expression of CRABP2 in tumor Schwann cells makes these cells principally suitable for ATRA treatment.

In a previous study the transcriptional co-activator ZNF423 was suppressed in neuroblastoma cells lacking NF1 due to overactive MAPK cascade signaling, finally leading to ATRA resistance [41]. Inhibiting the cascade by MEKi treatment led to ATRA response [42]. Thus, a mutual factor of ATRA and Ras signaling was identified by this group. Although in our study ATRA and MEKi treatment induced enhanced therapeutic effects, this was not attributed to changes of *ZNF423* mRNA levels. *ZNF423* levels did not correlate at all with ATRA sensitivity of MPNST. This is in obvious contrast to previous studies and demonstrates other mechanisms involved in MPNST cells compared to neuroblastoma cells.

Our data point to a potentially successful treatment of MPNST by combined application of ATRA and MEK inhibitors such as U0126 or PD0325901. In further experiments, effects other than reduced cellular proliferation of this combination therapy should be monitored to understand the biological impact. This therapeutic strategy utilizes two specific characteristics of NF1 associated MPNST—first, over active MAPK signaling and second, CRABP2 expression of these tumors. Additive effects on reduced viability were successfully demonstrated in two MPNST cells lines, whereas a probable resistance mechanism in ATRA/MEKi resistant NSF1 cells needs further investigations. Since NF1 deficient tumor cells are generally characterized by over-activation of Ras and, as we demonstrated, by CRABP2 expression, the combination of MEKi and ATRA seems to be a promising new approach, necessarily to be validated in animal experiments.

## Supporting information

S1 Fig Expression profile of nuclear receptors. MPNST cell lines (NSF1, S462, T265), normal human fibroblasts (nFib) and normal human Schwann cells (nhSC) were analyzed by PCR. Negative control conditions are labelled with H_2_O. All six receptor subtypes were demonstrated to be expressed on mRNA level in each of the analyzed cell types, except the *RXRG* receptor that was not present in NSF1 cells (one representative image is shown of n = 3).
(PDF)

S2 Fig Genomic structure of the CRABP2 gene (S2A), methylation analysis of CRBP2 promoter by prosequencing (S2B), and CRABP2 promoter methylation profiles of MPNST cells under ATRA treatment (S2C). S2A: Schematic map of the CRABP2 gene, including 5`CpG island and its relative orientation to the transcription start point (exons = filled boxes, UTR = open boxes, transcription initiation site = +1). The 129 single CpG sites of the whole CpG island are illustrated as single vertically dashes in the lower part. Analyzed sequences within the CpG island are indicated below the single CpG sites. S2B: Methylation profiles of normal human Schwann cells (nhSC) and MPNST cell lines were demonstrated for three analyzed regions of the CpG island, with mean CpG methylation in % for each CpG (SD < 7% is not shown). Exact number of each CpG site analyzed was depicted in the bottom line (grey box). Methylation profiles differed highly between the MPNST cell lines. T265 cell line showed similar methylation pattern (max. methylation per CpG site < 16.9%) to nhSC control cells. S462 cells demonstrated high methylation status for all CpG sites (mean methylation of 71.0%). The NSF1 cell line showed a highly variable methylation pattern with methylation status ranging from 3.4% to 72.0% for single CpG sites (mean, n = 4). S2C: No differences were observed in relative methylation status (%) of all analyzed CpG sites in ATRA treated MPNST cells compared to untreated cells (mean ± SD, n = 4).
(PDF)

S3 Fig Flow cytometry analysis of MPNST cell lines treated with ATRA. Relative increase of size (FSC, light grey) and granularity (SSC, dim grey) is given in % compared to untreated controls (0%). Relative cell size was increased by 16% in NSF1 cells, 14% in S462 cells and 6% in T265 cells. Granularity was increased by 14% in T265 cells, 22% in S462 cells and 39% in NSF1 cells (p<0.05, one-sided t-test, mean + SD, n = 3).
(PDF)

S4 Fig Apoptosis (TUNEL) staining in ATRA treated T265 cells. Merged images of DAPI and TUNEL are depicted for MPNST cell line T265. Number of TUNEL positive cell nuclei is clearly increased in ATRA treated cultures as compared to controls (exemplarily shown images of immunocytochemistry staining).
(PDF)

S5 Fig Relative mRNA levels in MPNST cells by qRT-PCR. PDK1 expression was not affected in MPNST cells treated with ATRA (grey bars) as compared to untreated cells (black line). FABP5 expression was not affected by ATRA treatment in S462 cells and NSF1 cells, and only slightly induced in T265 cells, as compared to untreated control cells (black line, 1) (mean + SD, n = 3).
(PDF)

S6 Fig Relative mRNA expression of CRABP2 and ZNF423 after MEKi treatment in MPNST cells by qRT-PCR. MPNST cells were incubated with different doses of PD0325901. CRABP2 expression was found to be induced at all concentrations in T265 and S462 cells (grey bars) compared to untreated control cells (black line). NSF1 cells showed decreased CRABP2 level at 1 nM and 10 nM PD0325901, but increased level at 1000 nM. ZNF423 expression was reduced in T265 cells in a dose-dependent manner but was not affected in S462 cells at all concentrations. Reduced ZNF423 levels were also found in NSF1 cells. Relative mRNA level were not determined in T265 cells at 1000 nM PD0325901, since almost no alive cells were present (n.d. = not determined) (mean + SD, n = 3).
(PDF)

S7 Fig Relative mRNA expression in MPNST cell lines after combined treatment with ATRA and PD by qRT-PCR. MPNST cells were treated with ATRA and MEKi PD0325901 alone or with a combination (light colored, dark colored and striped colored bars, respectively) (2 d). CRABP2, CYP26A1 and RARB mRNA expression were induced in all MPNST cell lines. Mild additive effects on induction of CRABP2 mRNA expression via combined therapy were observed in T265 and NSF1 cells compared to mono-therapy (mean + SD, n = 3).
(PDF)

S8 Fig Concentrations, primer and antibody specifications. Concentrations of pharmaceutical agents used for combination treatment (Table A). Primer sequences for RT-PCR (Table B). Primer sequences used for bisulfite-sequencing (Table C). Primer sequences used for amplification of bisulfite converted DNA (Table D). Specifications of antibodies used for western blot analysis (Table E).
(PDF)
