# Supplementary material for: MEK inhibitors enhance therapeutic response towards ATRA in NF1 associated malignant peripheral nerve sheath tumors (MPNST) in-vitro
Source: PLoS One. 2017 Nov 13;12(11):e0187700. doi: 10.1371/journal.pone.0187700 (PMC5683628; doi:10.1371/journal.pone.0187700)

Supporting Information

S1 Fig.: Expression profile of nuclear receptors.

MPNST cell lines (NSF1, S462, T265), normal human fibroblasts (nFib) and normal human Schwann cells (nhSC) were analyzed. Negative control conditions are labelled with H2O. All six receptor subtypes were demonstrated to be expressed on mRNA level in each of the analyzed cell types, except the RXRG receptor that was not present in NSF1 cells (one representative image is shown of n =3).

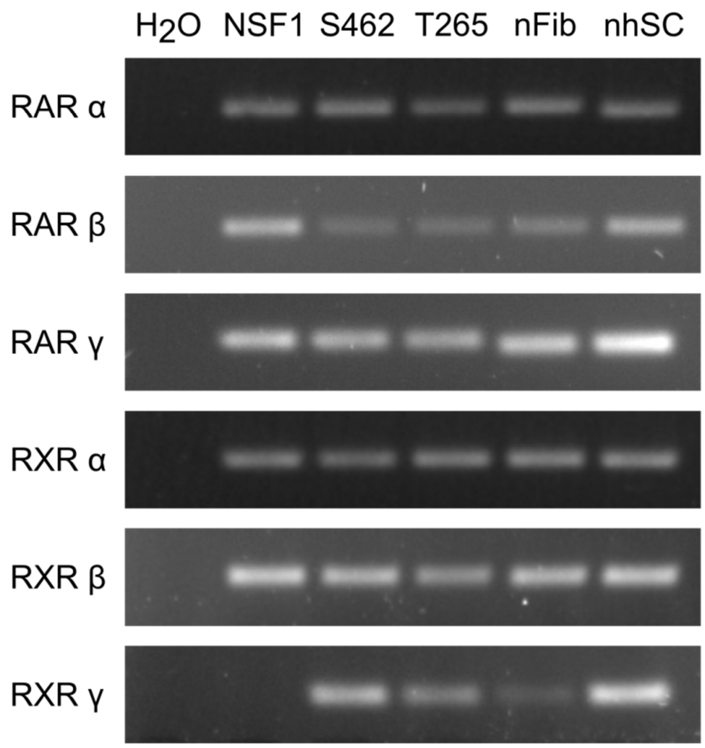

Supplement: S1 Fig — MPNST cell lines (NSF1, S462, T265), normal human fibroblasts (nFib) and normal human Schwann cells (nhSC) were analyzed by PCR. Negative control conditions are labelled with H2O. All six receptor subtypes were demonstrated to be expressed on mRNA level in each of the analyzed cell types, except the RXRG receptor that was not present in NSF1 cells (one representative image is shown of n = 3). (PDF) [file pone.0187700.s001.pdf]
