# Supplementary material for: MEK inhibitors enhance therapeutic response towards ATRA in NF1 associated malignant peripheral nerve sheath tumors (MPNST) in-vitro
Source: PLoS One. 2017 Nov 13;12(11):e0187700. doi: 10.1371/journal.pone.0187700 (PMC5683628; doi:10.1371/journal.pone.0187700)
Supplement: S2 Fig — S2A: Schematic map of the CRABP2 gene, including 5`CpG island and its relative orientation to the transcription start point (exons = filled boxes, UTR = open boxes, transcription initiation site = +1). The 129 single CpG sites of the whole CpG island are illustrated as single vertically dashes in the lower part. Analyzed sequences within the CpG island are indicated below the single CpG sites. S2B: Methylation profiles of normal human Schwann cells (nhSC) and MPNST cell lines were demonstrated for three analyzed regions of the CpG island, with mean CpG methylation in % for each CpG (SD < 7% is not shown). Exact number of each CpG site analyzed was depicted in the bottom line (grey box). Methylation profiles differed highly between the MPNST cell lines. T265 cell line showed similar methylation pattern (max. methylation per CpG site < 16.9%) to nhSC control cells. S462 cells demonstrated high methylation status for all CpG sites (mean methylation of 71.0%). The NSF1 cell line showed a highly variable methylation pattern with methylation status ranging from 3.4% to 72.0% for single CpG sites (mean, n = 4). S2C: No differences were observed in relative methylation status (%) of all analyzed CpG sites in ATRA treated MPNST cells compared to untreated cells (mean ± SD, n = 4). (PDF) [file pone.0187700.s002.pdf]

Supporting Information

S2 Fig: Genomic structure of the *CRABP2* gene (S2A), methylation analysis of CRBP2 promoter by prosequencing (S2B), and *CRABP2* promoter methylation profiles of MPNST cells under ATRA treatment (S2C)

S2A: Schematic map of the CRABP2 gene, including 5`CpG island and its relative orientation to the transcription start point (exons = filled boxes, UTR = open boxes, transcription initiation site = +1). The 129 single CpG sites of the whole CpG island are illustrated as single vertically dashes in the lower part. Analyzed sequences within the CpG island are indicated below the single CpG sites.

S2B: Methylation profiles of normal human Schwann cells (nhSC) and MPNST cell lines were demonstrated for three analyzed regions of the CpG island, with mean CpG methylation in % for each CpG (SD < 7% is not shown). Exact number of each CpG site analyzed was depicted in the bottom line (grey box). Methylation profiles differed highly between the MPNST cell lines. T265 cell line showed similar methylation pattern (max. methylation per CpG site < 16.9 %) to nhSC control cells. S462 cells demonstrated high methylation status for all CpG sites (mean methylation of 71.0 %). The NSF1 cell line showed a highly variable methylation pattern with methylation status ranging from 3.4 % to 72.0 % for single CpG sites (mean, n = 4).

S2C: No differences were observed in relative methylation status (%) of all analyzed CpG sites in ATRA treated MPNST cells compared to untreated cells (mean ± SD, n = 4).

2A

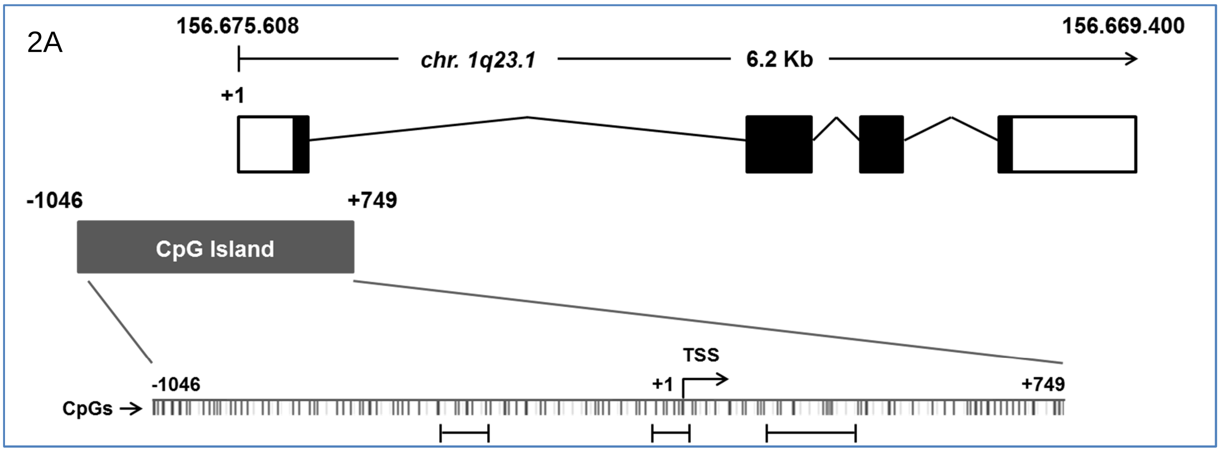

2B

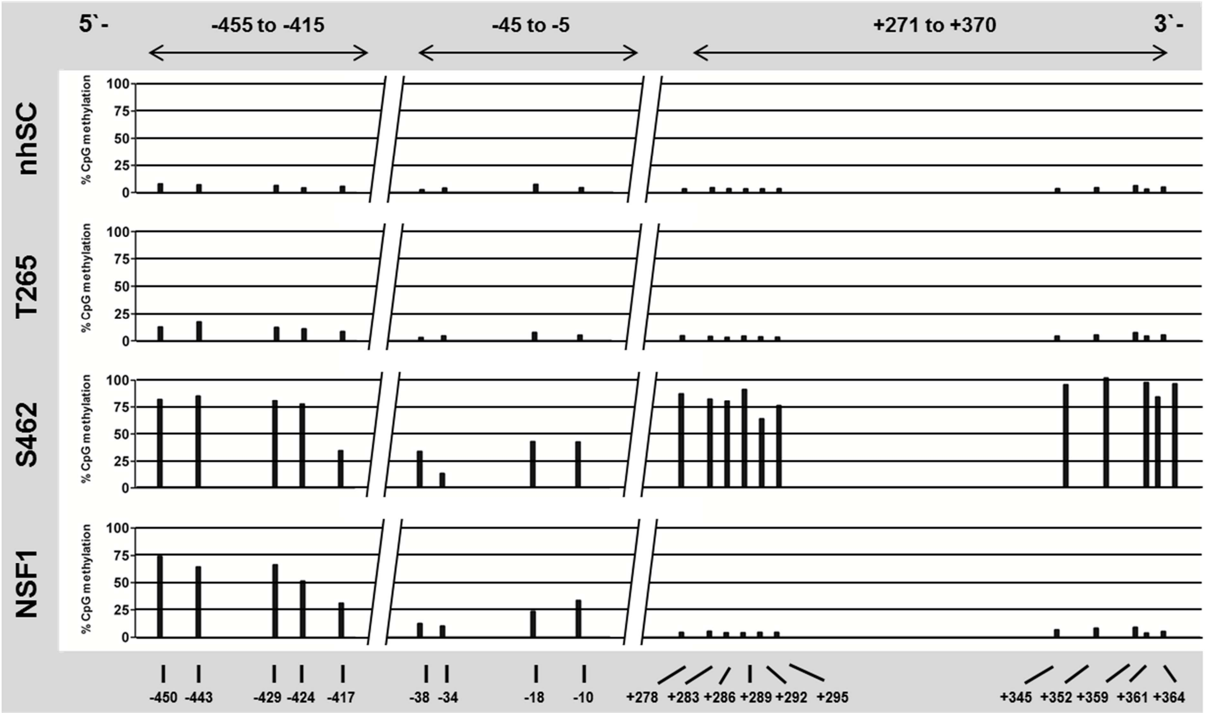

5`-
-455 to -415
-45 to -5
+271 to +370
3`-
nhSC
T265
S462
NSF1
% CpG methylation
100
75
50
25
0
-450 -443 -429 -424 -417 -38 -34 -18 -10 +278 +283 +286 +289 +292 +295 +345 +352 +359 +361 +364

2C

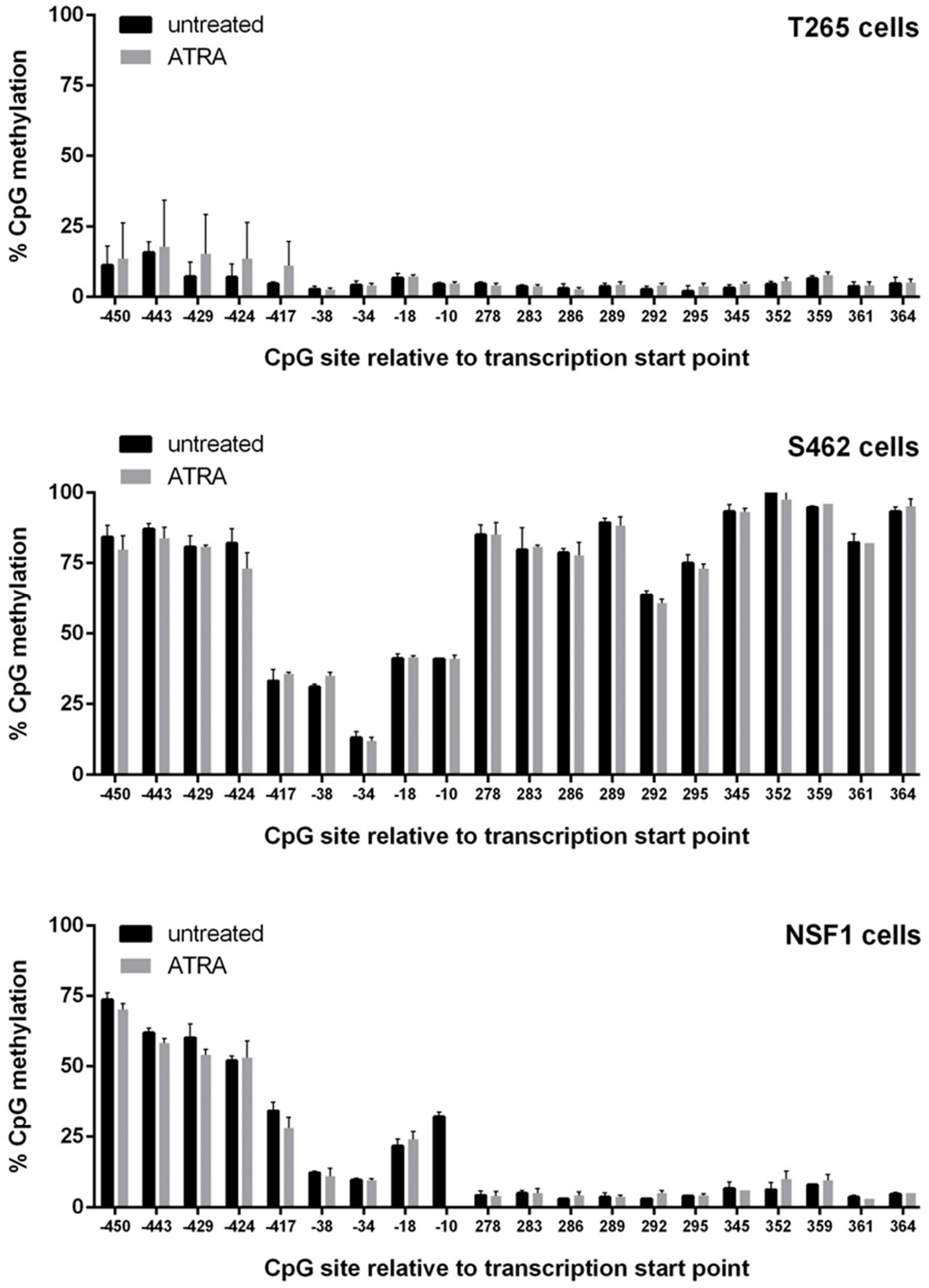
T265 cells
untreated
ATRA
% CpG methylation
100
75
50
25
0
-450
-443
-429
-424
-417
-38
-34
-18
-10
278
283
286
289
292
295
345
352
359
361
364
CpG site relative to transcription start point
S462 cells
NSF1 cells
