# Supplementary material for: MEK inhibitors enhance therapeutic response towards ATRA in NF1 associated malignant peripheral nerve sheath tumors (MPNST) in-vitro
Source: PLoS One. 2017 Nov 13;12(11):e0187700. doi: 10.1371/journal.pone.0187700 (PMC5683628; doi:10.1371/journal.pone.0187700)

Supporting Information

S3 Fig.: Flow cytometry analysis for FSC and SSC of three MPNST cell lines treated with ATRA.

Relative increase of size (FSC, light grey) and granularity (SSC, dim grey) is given in % compared to untreated controls (0%). Relative cell size was increased by 16% in NSF1 cells, 14% in S462 cells and 6% in T265 cells. Granularity was increased by 14% in T265 cells, 22% in S462 cells and 39% in NSF1 cells ($p<0.05$, one-sided t-test, mean + SD, n = 3).

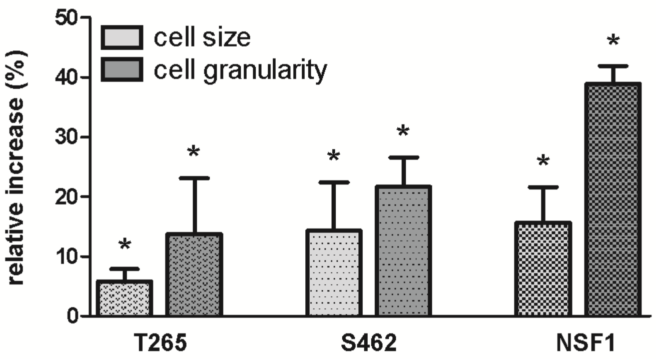

Supplement: S3 Fig — Relative increase of size (FSC, light grey) and granularity (SSC, dim grey) is given in % compared to untreated controls (0%). Relative cell size was increased by 16% in NSF1 cells, 14% in S462 cells and 6% in T265 cells. Granularity was increased by 14% in T265 cells, 22% in S462 cells and 39% in NSF1 cells (p<0.05, one-sided t-test, mean + SD, n = 3). (PDF) [file pone.0187700.s003.pdf]
