# Supplementary material for: MEK inhibitors enhance therapeutic response towards ATRA in NF1 associated malignant peripheral nerve sheath tumors (MPNST) in-vitro
Source: PLoS One. 2017 Nov 13;12(11):e0187700. doi: 10.1371/journal.pone.0187700 (PMC5683628; doi:10.1371/journal.pone.0187700)

Supporting Information

S4 Fig.: Apoptosis (TUNEL) staining in ATRA treated T265 cells.

Merged images of DAPI and TUNEL are depicted for MPNST cell line T265. Number of TUNEL positive cell nuclei is clearly increased in ATRA treated cultures as compared to controls (exemplarily shown images).

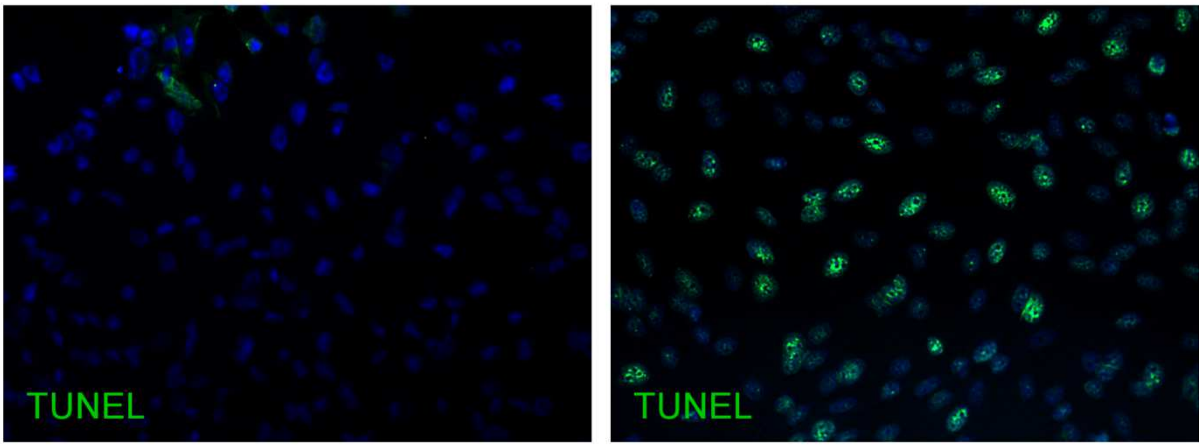

Supplement: S4 Fig — Merged images of DAPI and TUNEL are depicted for MPNST cell line T265. Number of TUNEL positive cell nuclei is clearly increased in ATRA treated cultures as compared to controls (exemplarily shown images of immunocytochemistry staining). (PDF) [file pone.0187700.s004.pdf]
