# Supplementary material for: MEK inhibitors enhance therapeutic response towards ATRA in NF1 associated malignant peripheral nerve sheath tumors (MPNST) in-vitro
Source: PLoS One. 2017 Nov 13;12(11):e0187700. doi: 10.1371/journal.pone.0187700 (PMC5683628; doi:10.1371/journal.pone.0187700)

Supporting Information

S5 Fig.: Relative mRNA levels in MPNST cells by qRT-PCR.

PDK1 expression was not affected in MPNST cells treated with ATRA (grey bars) as compared to untreated cells (black line). FABP5 expression was not affected by ATRA treatment in S462 cells and NSF1 cells, and only slightly induced in T265 cells, as compared to untreated control cells (black line, 1) (mean + SD, n = 3).

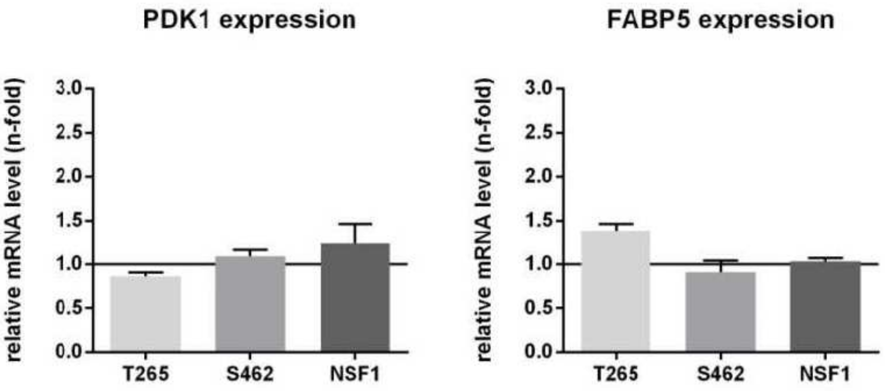

Supplement: S5 Fig — PDK1 expression was not affected in MPNST cells treated with ATRA (grey bars) as compared to untreated cells (black line). FABP5 expression was not affected by ATRA treatment in S462 cells and NSF1 cells, and only slightly induced in T265 cells, as compared to untreated control cells (black line, 1) (mean + SD, n = 3). (PDF) [file pone.0187700.s005.pdf]
