# Supplementary material for: MEK inhibitors enhance therapeutic response towards ATRA in NF1 associated malignant peripheral nerve sheath tumors (MPNST) in-vitro
Source: PLoS One. 2017 Nov 13;12(11):e0187700. doi: 10.1371/journal.pone.0187700 (PMC5683628; doi:10.1371/journal.pone.0187700)

**Supporting Information**

**S6 Fig.: Relative mRNA expression of CRABP2 and ZNF423 after MEKi treatment in MPNST cells by qRT-PCR.**

MPNST cells were incubated with different doses of PD0325901. CRABP2 expression was found to be induced at all concentrations in T265 and S462 cells (grey bars) compared to untreated control cells (black line). NSF1 cells showed decreased CRABP2 level at 1 nM and 10 nM PD0325901, but increased level at 1000 nM. ZNF423 expression was reduced in T265 cells in a dose-dependent manner but was not affected in S462 cells at all concentrations. Reduced ZNF423 levels were also found in NSF1 cells. Relative mRNA level were not determined in T265 cells at 1000 nM PD0325901, since almost no alive cells were present (n.d. = not determined) (mean + SD, n = 3).

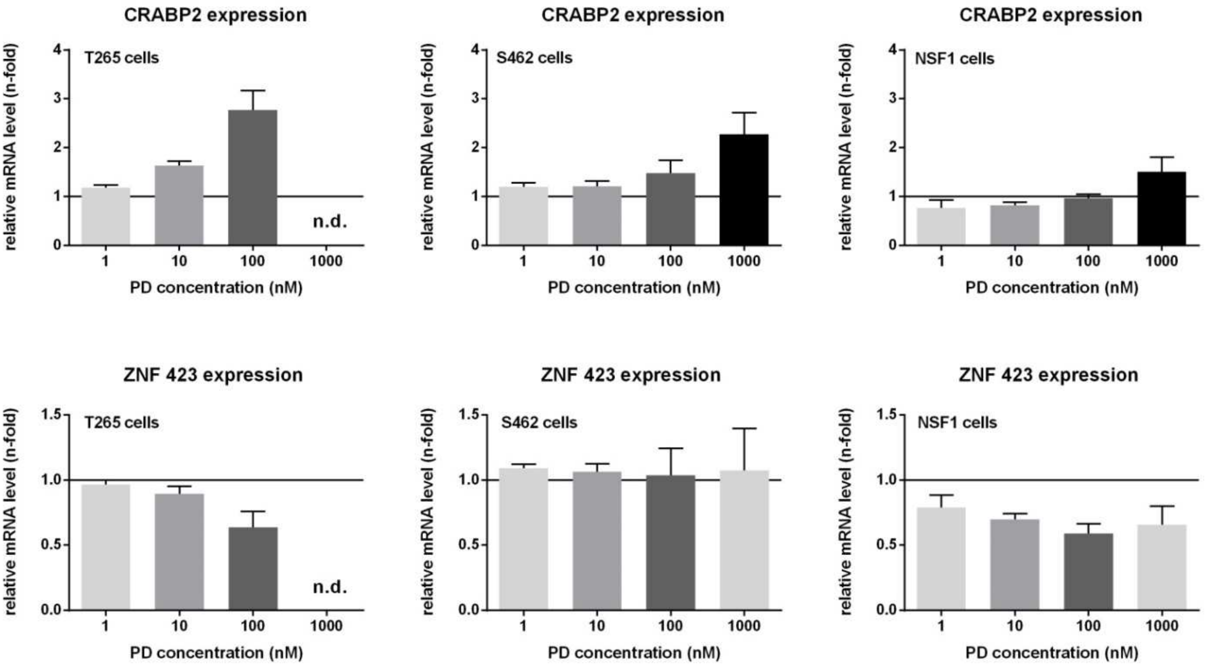

Supplement: S6 Fig — MPNST cells were incubated with different doses of PD0325901. CRABP2 expression was found to be induced at all concentrations in T265 and S462 cells (grey bars) compared to untreated control cells (black line). NSF1 cells showed decreased CRABP2 level at 1 nM and 10 nM PD0325901, but increased level at 1000 nM. ZNF423 expression was reduced in T265 cells in a dose-dependent manner but was not affected in S462 cells at all concentrations. Reduced ZNF423 levels were also found in NSF1 cells. Relative mRNA level were not determined in T265 cells at 1000 nM PD0325901, since almost no alive cells were present (n.d. = not determined) (mean + SD, n = 3). (PDF) [file pone.0187700.s006.pdf]
