# Supplementary material for: MEK inhibitors enhance therapeutic response towards ATRA in NF1 associated malignant peripheral nerve sheath tumors (MPNST) in-vitro
Source: PLoS One. 2017 Nov 13;12(11):e0187700. doi: 10.1371/journal.pone.0187700 (PMC5683628; doi:10.1371/journal.pone.0187700)

Supporting Information

S7 Fig: Relative mRNA expression in MPNST cell lines after combined treatment with ATRA and PD0325901 by qRT-PCR.

MPNST cells were treated with ATRA and MEKi PD0325901 alone or with a combination (light colored, dark colored and striped colored bars, respectively) (2 d). CRABP2, CYP26A1 and RARB mRNA expression were induced in all MPNST cell lines. Mild additive effects on induction of CRABP2 mRNA expression via combined therapy were observed in T265 and NSF1 cells compared to mono-therapy (mean + SD, n = 3).

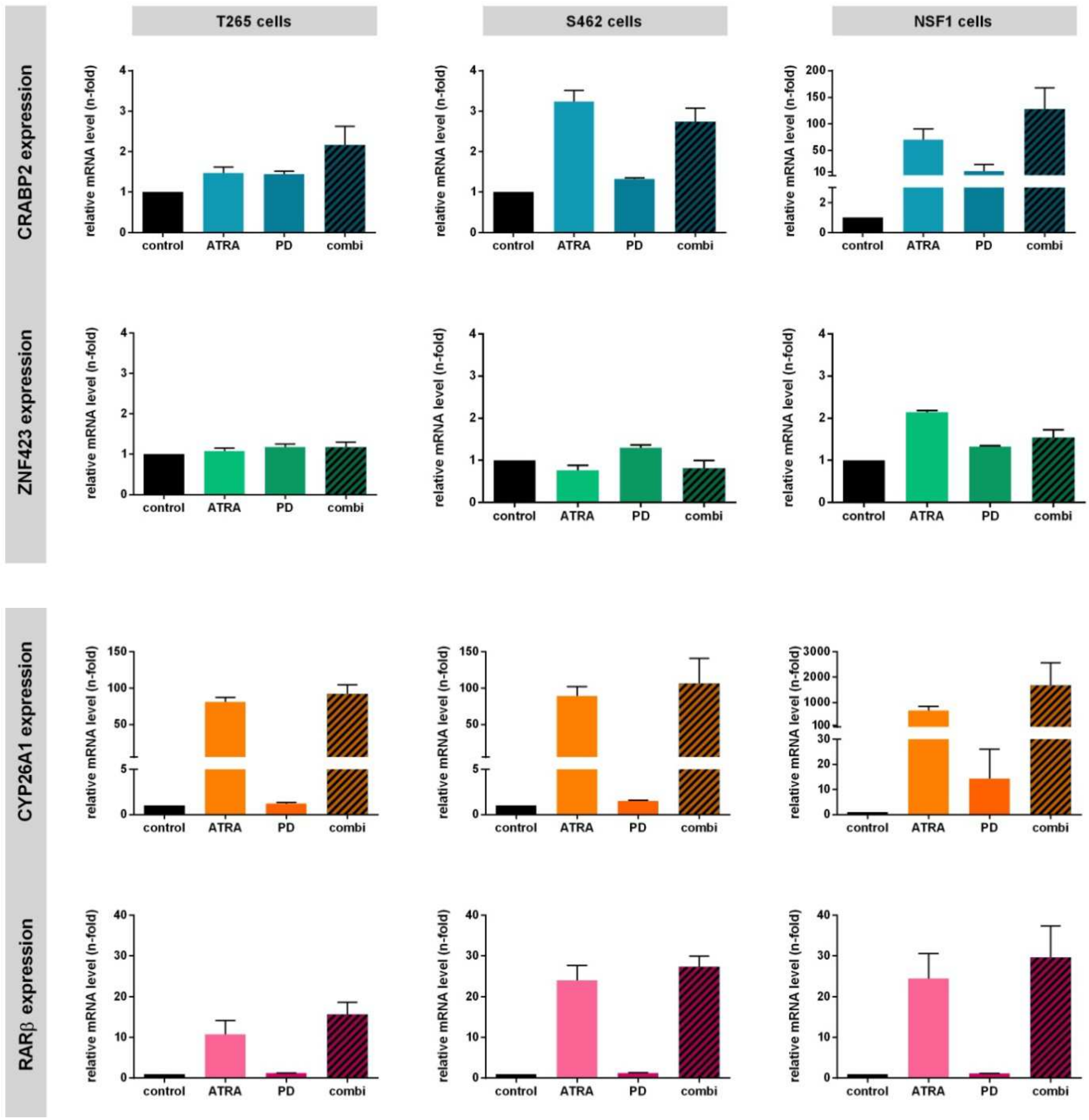

Supplement: S7 Fig — MPNST cells were treated with ATRA and MEKi PD0325901 alone or with a combination (light colored, dark colored and striped colored bars, respectively) (2 d). CRABP2, CYP26A1 and RARB mRNA expression were induced in all MPNST cell lines. Mild additive effects on induction of CRABP2 mRNA expression via combined therapy were observed in T265 and NSF1 cells compared to mono-therapy (mean + SD, n = 3). (PDF) [file pone.0187700.s007.pdf]
