# Supplementary material for: MEK inhibitors enhance therapeutic response towards ATRA in NF1 associated malignant peripheral nerve sheath tumors (MPNST) in-vitro
Source: PLoS One. 2017 Nov 13;12(11):e0187700. doi: 10.1371/journal.pone.0187700 (PMC5683628; doi:10.1371/journal.pone.0187700)
Supplement: S8 Fig — Concentrations of pharmaceutical agents used for combination treatment (Table A). Primer sequences for RT-PCR (Table B). Primer sequences used for bisulfite-sequencing (Table C). Primer sequences used for amplification of bisulfite converted DNA (Table D). Specifications of antibodies used for western blot analysis (Table E). (PDF) [file pone.0187700.s008.pdf]

Supporting Information - S8 Fig

Table A:
Concentrations of pharmaceutical agents used for combination treatment

	**T265 cells**	**S462 cells**	**NSF1 cells**
**U0126**	2.5 µM	2.5 µM	10 µM
**PD0325901**	10 nM	10 nM	100 nM
**ATRA**	25 µM	10 µM	50 µM

Table B:
Primer sequences for RT-PCR

F - forward, R - reverse, P – probe; PCR primer and conditions were optimized in order to guarantee one single PCR product for each PCR reaction. Amplicon size was verified by resolving on agarose gels for conventional PCR*. Single amplicons were verified by melting curve analysis after quantitative PCR#. PCR via manufactured TaqMan probes did not require amplicon verification°.

**Gene**		**Sequence (5`→ 3`)**	**Amplicon length (bp)**
GAPDH#	F	acccactcctccacctttgac	76
	R	cataccaggaaatgagcttgacaa	
	P	ctggcattgccctcaacgacca	
CRABP2°		Gene Expression Assay Applied Biosystems: Hs00275636_m1	not specified
FABP5°		Gene Expression Assay Applied Biosystems: Hs02339439_g1	not specified
ZNF423°		Gene Expression Assay Applied Biosystems: Hs00323880_m1	not specified
CYP26A1#	F	ctggacatgcaggcactaaa	100
	R	gccccaggtaagtgatcaga	
PDK1#	F	ggttacgggacagatgcagt	109
	R	cgtggttggtgttgtaatgc	
RARA*	F	ggcatgtccaaggagtctgtg	90
	R	cgtcagcgtgtagctctcag	
RARB#	F	ttctcagtgccatctgcttaatct	130
	R	ggcttgctgggtcgtcttttt	
RARG*	F	aacaaggtgaccaggaatcg	133
	R	tgtcaggtgacccttcttcc	
RXRA*	F	caaggactgcctgattgaca	150
	R	ctggtcgactccacctcatt	
RXRB*	F	tcagcagcccccagattaac	151
	R	attgcacatagccgtttgcc	
RXRG*	F	cgggattggaaacatgaact	103
	R	taccccgtagtgctttcctg	

[illegible]

Table C:
Primer sequences used for bisulfite-sequencing

**Sequence (relative to TSS)**	**Sequence (5`→ 3`) methylated**	**Sequence (5`→ 3`) un-methylated**
-450 to -416	tcctgcttaacccttcaatgtc	ttttgtttaattttttaatgtt
-38 to +1	acacgagccctgagcatctg	atatgagttttgagtatttg
+277 to +299	tcttggttccagaacctg	ttttggttttagaatttg
+344 to +365	gtgctccagcctaggagtcta	gtgttttagtttaggagttta

Table D:
Primer sequences used for amplification of bisulfite converted DNA
(F – forward, R – reverse)

**Sequence (relative to TSS)**		**Sequence (5`→ 3`) methylated**	**Sequence (5`→ 3`) un-methylated**
-450 to -416	F	aataaagauuttgcgtcaagtgattg	aataaagattttgtgtcaagtgattg
	R	actcacgtaatacctaaagactctaaaaa	tttttagagtctttaggtattacgtgagt
-38 to +1	F	gaattcgggtgggggatagttatata	gaatttgggtgggggatagttatata
	R	ccaccaacctctaaatctaaccc	gggttagatttagaggttggtgg
+277 to +365	F	gggagtttaggttagttttgggg	gggagtttaggttagttttgggg
	R	cccaacactttaaacaattcctc	gaggaattgutuaaagtgutggg

Table E:
Specifications of antibodies used for western blot analysis

**Antibody**	**Specification**	**Species**	**Dilution**	**Manufacturer**
α-CRABP2	polyclonal	rabbit	1:250	Abcam (ab74365)
α-β-actin	monoclonal	mouse	1:200000	Cell Signaling (#3700)
α-GAPDH	polyclonal	rabbit	1:10000	Sigma (G9545)
α-ERK	monoclonal	rabbit	1:2000	Cell Signaling (#4696)
α-p-ERK	monoclonal	rabbit	1:4000	Cell Signaling (#4370)
α-Rabbit	monoclonal, hrp-coupled	mouse	1:10000	Sigma (A 2074)
α-Mouse	polyclonal, hrp-coupled	goat	1:10000	Sigma (A3682)
